# Noble Gases in Solid Compounds Show a Rich Display of Chemistry With Enough Pressure

**DOI:** 10.3389/fchem.2020.570492

**Published:** 2020-11-05

**Authors:** Maosheng Miao

**Affiliations:** Department of Chemistry and Biochemistry, California State University Northridge, Northridge, CA, United States

**Keywords:** noble gas anions, NG–NG bonds, noble gas bond, chemistry without chemical bond, DFT—density functional theory, high pressure

## Abstract

In this review, we summarize the rapid progress that has been made in the study of noble gas chemistry in solid compounds under high pressure. Thanks to the recent development of first-principles crystal structure search methods, many new noble gas compounds have been predicted and some have been synthesized. Strikingly, almost all types of chemical roles and interactions are found or predicted in these high-pressure noble gas compounds, ranging from cationic and anionic noble gases to covalent bonds between noble gas atoms, and to hydrogen bond-like noble gas bonds. Besides, the recently discovered He insertion reactions reveal a unique chemical force that displays no local chemical bonding, providing evidence that research into noble gas reactions can advance the frontier of chemistry at the very basic level.

## Introduction

For many years after their discovery, noble gases (NG) were known as elements that do not have any chemistry. This idea was consolidated by the atomic shell structure and the corresponding theory that all elements are destined to a complete shell while forming compounds. Therefore, the noble gases would remain chemically inert (noble) since their valence orbitals are already completely filled. This doctrine was first challenged in 1933 by Pauling who predicted the formation of KrF_6_ and XeF_6_ compounds (Pauling, 1933). It took almost another 30 years before the first noble gas compound, XePtF_6_ was synthesized by Bartlett (1962). Soon after, many Xenon binary compounds such as XeF_4_, XeF_2_ etc. were obtained (Chernick et al., 1962). By now, there are already a few hundred known noble gas compounds and the list continues to grow (Grochala, 2007). The most recent advancements include the first truly bonded Argon compound, HArF (Khriachtchev et al., 2000), and a striking compound AuXe_4_ (Sb_2_F_11_) (Seidel and Seppelt, 2000) in which Xenon, a noble gas element, bonds with Au, a noble metal, as a weak reducing and coordinating agent.

A scenario of rich chemistry for NG elements has been rolled out gradually over the past decades, especially while locking them in an extreme chemical environment. For example, many NG elements can be coerced to form charged or strongly polarized species, such as HHe^+^ (Hogness and Lunn, 1925), HNGO^−^ (Li et al., 2005), and HeOLi_2_F_2_, etc. (Grochala, 2012). In contrast, a similar chemical environment is difficult to achieve in solid compounds since the charge neutrality needs to be preserved globally and locally. As a result, the chemical roles that NG elements can play in solid compounds are more limited. Lots of noble gas compounds are not formed by local chemical bonds featuring electron sharing or transfer. In many of these compounds, noble gases are either bonded to other atoms by weak interactions such as van der Waals force or inserted into the voids preexisting in some solid compounds such as clathrates, C_60_, etc. (Saunders et al., 1994; Guńka et al., 2015). While forming true chemical bonds in compounds, such as XeF_2_, XeO_3_, etc., noble gases act like reductants (electron donors).

On the other hand, we can drive chemical interactions to an extreme in solid compounds by applying mechanical pressures so that new chemistry can emerge. Due to both the development of first-principles computer simulations (Zhang et al., 2017; Oganov et al., 2019; Miao et al., 2020) and the diamond anvil cell (DAC) experiments (Mao et al., 2018), numerous novel compounds have been predicted and some have been synthesized. These new compounds under pressure, such as Na_m_Cl_n_ (Zhang et al., 2013), H_3_S (Drozdov et al., 2015), LaH_n_ (Pickard et al., 2020), CsF_n_ (Miao, 2013), etc., show a distinct trend of having a large range of compositions, with very different stoichiometries to the ambient condition. Although many unconventional stoichiometries are caused by the formation of homonuclear bonds or species, such as Cl–Cl in NaCl_3_, some compounds with atypical compositions are formed due to the change of oxidation states of their constituent elements (Miao et al., 2020). In some extreme cases, the core electrons, such as the 5p electrons of Cs, can be coerced to form chemical bonds, leading to the formation of atypical compounds, such as CsF_3_ and CsF_5_ (Miao, 2013).

For the same reason, pressure can greatly enrich noble gas chemistry. It goes far beyond the known NG compounds formed by sharing their closed-shell electrons with strong oxidants such as F. In contrast to molecular and ionic species, most of the solid NG compounds under pressure are thermodynamically stable. In this review, we will show that NG elements, under high-pressure, can (1) be oxidized by elements such as Fe that usually are not considered as oxidants, (2) become an oxidant themselves and behave like anions in compounds, (3) form strong NG–NG covalent bonds, (4) form intermolecular NG bonds that are similar to hydrogen bonds, and (5) form stable compounds that are not bound by any local chemical bonds.

## Methods and the Progress of High-Pressure Chemistry

The progresses of high-pressure chemistry strongly depend on the development of experimental methods. The first leap of this field is triggered by the development of diamond anvil cell (DAC) and the corresponding heating and measurement techniques. However, high-pressure experiments are usually very difficult, expensive, and time-consuming. Recently, the density functional theory (DFT) calculations have been widely used in predicting phase diagrams of binary compounds under pressure, which gave rise to the second leap of high-pressure chemistry. These “complete ab initio” studies neither use any empirical parameters for electronic structure nor take any crystal structures and chemical bonding information as input. Instead, crystal structures are generated and the globally stable structures are searched, using various algorithms, such as random search (RS) (Pickard and Needs, 2011), genetic algorithm (GA) (Glass et al., 2006; Avery et al., 2019) and particle swarm optimization (PSO) (Wang et al., 2012). In the last decade, numerous new compounds have been predicted by this method without any experimental input and many of them have been confirmed by DAC experiments. Many of these compounds assume atypical compositions, such as NaCl_3_, CsF_3_, H_3_S, LaH_10_, etc. The change of the chemistry and the formation of a plethora of atypical compounds can be roughly grouped into two kinds: those caused by the formation of homonuclear bonds and those caused by the change of oxidation states, both of which can be found in high-pressure noble gas compounds (Miao et al., 2020).

## Noble Gas Chemistry Under High Pressure

### Oxidation of NG Under Pressure

Although most of noble gas chemistry is about the sharing of their closed-shell electrons, oxidizing NG is not an easy task and most of the stable NG compounds contain F, the strongest oxidant element. Several Xenon oxides exist but they are not stable (Brock and Schrobilgen, 2011; Goettel et al., 2016). Pressure can greatly extend the chemistry of NG as a reductant because the energies of their valence orbitals increase rapidly under pressure and become significantly higher than those of the valence orbitals of oxidant elements. For example, DFT/GA simulations showed that XeO, XeO_2_, and XeO_3_ become stable at pressures above 83, 102, and 114 GPa (Zhu et al., 2013). A study that combined the DAC experiment and DFT simulation work added two new compositions, Xe_3_O_2_ and Xe_2_O_5_, in which Xe adopted mixed oxidation states (Dewaele et al., 2016). Similarly, Kr–O (Zaleski-Ejgierd and Lata, 2016), Xe–N (Peng et al., 2015), and Xe–C (Bovornratanaraks et al., 2019) compounds are predicted by DFT/GA, DFT/PSO, and DFT/GA methods respectively. Probably the most striking prediction is the formation of stable Xe–Fe and Xe–Ni compounds under high pressure (Zhu et al., 2014), which has been confirmed by DAC experiments (Dewaele et al., 2017; Stavrou et al., 2018). Especially, the DFT/PSO calculations showed that XeFe_3_ and XeNi_3_ become stable at the pressures and temperatures found in the Earth's core, indicating that the iron core of the Earth might be a chemical reservoir of the missing Xe (Zhu et al., 2014). From the chemistry point of view, it is significant that Xe can be oxidized by Fe or Ni under high pressure, as shown by the large calculated charge transfer from Xe to Fe/Ni in these compounds. The alloying of Xe with transition metals such as Hg has been predicted before, but DFT calculations showed only a slight charge transfer from Xe to Hg (Grochala, 2007).

### Anionic Noble Gases Under Pressure

Besides extending the range of reductant chemistry, pressure can endow a new role for NG elements. They might oxidize metals such as Li and Mg and become anions in the corresponding compounds (Li et al., 2015; Miao et al., 2015; Liu et al., 2017). The first example was predicted by the DFT/PSO method, which showed that Mg forms stable compounds with Xe, Kr, and Ar under pressures higher than 125, 250, and 250 GPa, respectively (Miao et al., 2015). Among all the calculated compositions, MgNG and Mg_2_NG are stable under high pressure. These compounds adopt very simple structures. For Xe and Kr, MgNG adopts a Pm$\bar{3}$m (CsCl) structure, whereas Mg_2_NG adopts a P_4_/nmm structure. In contrast, both MgAr and Mg_2_Ar compounds adopt hexagonal P6_3_/mcm structures. The most important chemical feature of these compounds is the charge transfer. As calculated by Bader's Quantum Theory of Atoms in Molecules method (Bader, 1990), there are large charges transferred from Mg to NG, which also strongly depend on the pressure (Miao et al., 2015). For example, the charge transfer from Mg to Xe in MgXe under 100 GPa is 1.5 e/Mg, which is comparable to that in MgO under ambient conditions. From the band structures, these compounds are clearly metallic. The projected density of states (PDOS) reveals that the transferred electrons occupy the Xe 5d orbitals (Miao et al., 2015). Therefore, under high pressure, Xe behaves like a 5d transition metal. The electron localization function (ELF) (Silvi and Savin, 1994) calculations also show large values between Mg and NG, which is also the characteristic of intermetallic compounds. A similar phenomenon has not been found or predicted in any case without compression. The closest sign of anionic NG is a theoretical study that shows a positive electron affinity for Oganesson (Gaston et al., 2002), a synthetic element that has a half-life of about 1 ms.

**Figure 1:**
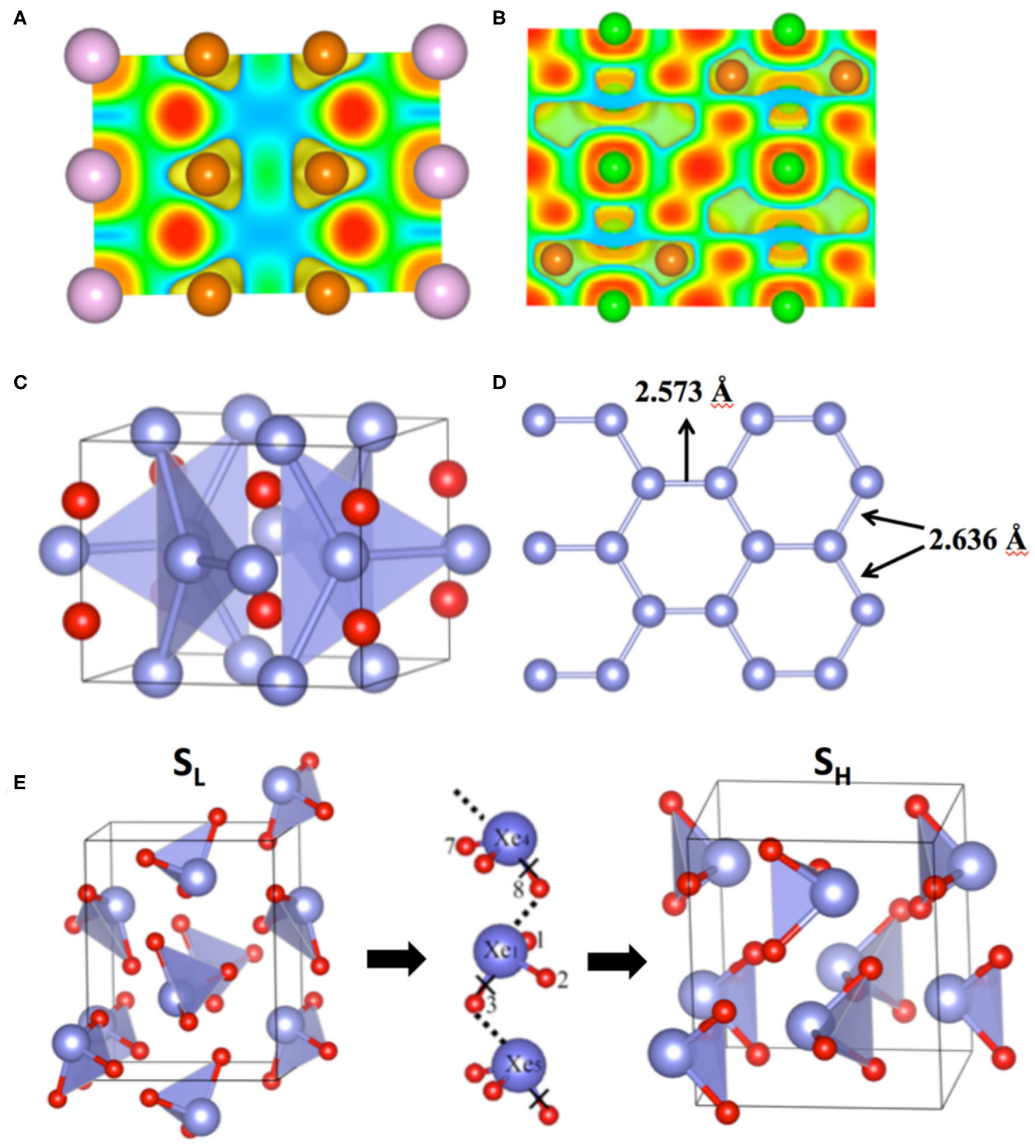
Electronic structure and geometry of compounds containing anionic noble gases, NG–NG covalent bonds and NG bonds. **(A)** Electron localization function (ELF) of Mg_2_Kr at 200 GPa; **(B)** ELF of Mg_2_Ar at 200 GPa; **(C)** Structure of Xe_2_F at 200 GPa; **(D)** The view of one set of Xe atoms in a graphene-like lattice in Xe_2_F. The bond lengths are slightly different due to the distortion. **(E)** The low pressure (S_L_) and the high pressure (S_H_) structures of XeO_3_ and the O path for the transition from S_L_ to S_H_ or vice versa. Brown, purple and green balls represent Mg, Xe and Ar atoms in **(A)** and **(B)**. Blue and red balls represent Xe and F (or O) atoms in **(C–E)**.

The Mg-rich compounds show another unique feature in their electronic structures. The charges on Mg and NG do not add up to 0. For example, at 50 GPa, the charges on Xe and Mg are −1.03e/Xe and 1.29e/Mg. There are about 1.54e which are not located on either Mg or Xe (Miao et al., 2015). Both the charge distribution and the ELF plots show that these charges locate at the interstitial sites between Mg and NG atoms (Figures 1A,B). Therefore, Mg_2_NG is a high-pressure electride (HPE). Electrides are compounds in which some electrons detach from all the atoms and locate at the interstitial sites, playing the role of anions (Dawes et al., 1986). The formation of electrides under high pressure can be explained by the energy change of a local orbital constrained at an interstitial site by the surrounding atoms (Miao and Hoffmann, 2014). Although its energy increases due to the reduced volume under increasing pressure, it may change less significantly than the energies of many atomic orbitals. A series of calculations of these orbital energy changes using a He-matrix model showed that the local orbital energy of the interstitial quasi-atoms (ISQ) decreases relative to that of s and p orbitals with a rate that strongly depends on the atom and the orbital (Miao and Hoffmann, 2014). HPE can form while the energy of ISQ becomes significantly lower than the energy of valence orbitals of the atom, for example, Li and Na. Mg metal has been predicted to become an HPE under pressures higher than 800 GPa (Li et al., 2010). The insertion of NG atoms into the Mg lattice while forming Mg–Xe compounds significantly lowers the pressure of forming HPE.

### NG–NG Covalent Bonds in Simple Compounds

The examples of chemical bonds between NG atoms are rare. In principle, they do not form bonds because their valence orbitals are completely filled. However, while one or both NG atoms lose electrons, they may form bonds. Examples of this kind include the Xe–Xe bonds in molecules HXeXeR and RXeXeR′ (R,R′=F, Cl, Br, I) (Fernández and Frenking, 2012), and the Xe^2+^ cations that occur in ${\text{Xe}}_{2}^{+}$(Sb_4_F_21_)^−^. (Drews and Seppelt, 1997) More recently, an example of the Xe–Xe bond was found unexpectedly in novel Xe–F compounds under pressure (Peng et al., 2016). An experimental study of XeF_2_ under compression showed that the XeF_2_ molecular crystal transformed into 2D and 3D extended solids and become metallic (Kim et al., 2010). However, the later DFT study did not agree with the proposed structure evolution of XeF_2_ under pressure, and therefore cannot explain the observed metallization of the compound (Kurzydlowski et al., 2011). This discrepancy was alleviated by a full-scale computation study of the Xe–F compounds with various compositions (Peng et al., 2016). The PSO based structure search revealed that XeF_2_ becomes unstable and decomposes to Xe_2_F and XeF_4_ at 81 GPa (Peng et al., 2016). DFT calculations using HSE functional show that XeF_2_ maintains its energy gap at least up to 100 GPa, whereas Xe_2_F is metallic. The observed insulator-metal transition of XeF_2_ at 70 GPa might be caused by the partial decomposition of the sample. The metallic transition was not observed in a later experiment where pressure is applied up to 80 GPa without heating the sample (Wu et al., 2017). Throughout its stable pressure range (60–200 GPa), Xe_2_F adopts an I4/mcm structure (Figure 1C) consisting of intercalated Xe graphitic (graphene-like) layers (Figure 1D) (Peng et al., 2016). At 200 GPa, the Xe–Xe distances are 2.573 and 2.636 Å (Figure 1D), which is close to the summation of the covalent radius of two Xe atoms. The calculated COHP and ELF prove that the two neighboring Xe atoms form covalent bonds (Peng et al., 2016). The appearance of Xe–Xe bonds in a simple binary compound is due to the enhancement of the homonuclear bond strength under pressure (Miao et al., 2020). It causes the instability of XeF_2_, a stoichiometric compound consisting of Xe in its typical oxidation state of +2.

### Noble Gas Bonds

Another type of chemical interaction that has been missing in noble gas chemistry is the donor-acceptor weak interaction between molecules, which is similar to hydrogen bonds (Pauling, 1960) Although this type of bond is the strongest for hydrogen, especially while H atoms are bonded with strong oxidant elements such as F and O and become very electrophilic, it has been found for other elements. It was extended to halogens where they are named halogen bonds (Cavallo et al., 2016), and then to chalcogens, pnictogens, etc. (Cavallo et al., 2016). Up till recently, almost all groups of elements in the periodic table have been found to form this type of bond, except noble gases. Recently, Bauzá and Frontera (2015) studied the molecular electrostatic potential surface of XeO_3_ and showed that there was an unexpectedly positive potential at the position of the lone pair of Xe^6+^, indicating that Xe are very electrophilic while in a high oxidation state and can form a noble gas bond. Similar bonding features are also found in XeO_3_ and alkylnitrile adducts (Goettel et al., 2016).

Under increasing pressure, molecular crystals bound by hydrogen bonds behave very differently to molecular crystals without it. In the latter case, the lengths of the covalent intramolecular bonds, such as C–H bonds in CH_4_, decrease while the intermolecular distances are reduced by pressure. In contrast, the lengths of some intramolecular bonds, such as H–O in H_2_O, increases under compression, if the hydrogen bonds dominate the intermolecular interactions. In accordance with this change, some vibration modes are softened by the external pressure, opposing our chemical intuition that all vibration frequencies should increase while the material is compressed. Therefore, the change of bond lengths and vibration modes under pressure can be used to demonstrate the presence of hydrogen bonds. The same idea can be applied to noble gas bonds. As shown by a recent computational work of Hou et al. (2017), under increasing pressure, the Xe–O bond lengths of both S_L_ and S_H_ increase, and the vibration frequencies of S_H_ decrease. Furthermore, the strong noble gas bonds between XeO_3_ molecules under pressure might provide transition paths for O atoms from one Xe to a neighboring Xe, a process that is essential for the structural transitions between S_L_ and S_H_ (Figure 1E) (Hou et al., 2015).

### Forming Compounds While Keeping Nobility

Being the second most abundant element in the universe, Helium has the highest ionization energy of 24.59 eV and a negative electron affinity. Thus, He shows much less chemistry than most other elements in the periodic table. Yet, several chemical species have been predicted or synthesized by locking He in an unusual chemical environment by exquisitely designed molecular scaffolding (Hogness and Lunn, 1925; Hotokka et al., 1984; Li et al., 2005; Rzepa, 2010; Grochala, 2012). In contrast, the chemistry of He in solid compounds is almost a blank slate except the insertion of He into solid compounds with clathrate or cage structures (Saunders et al., 1994; Guńka et al., 2015). Up till very recently, there is no known reaction of He that can form a stable solid compound. The first example of such is proposed by a thorough structure search study of elements in the periodic table reacting with He under pressure and confirmed by a DAC experiment. Most of the elements were found not to react with He except Na that will form a stable Na_2_He compound under pressure higher than 113 GPa (Dong et al., 2017) The enthalpy of formation is as large as a 0.35 eV/atom at a pressure of 350 GPa. Such a large energy gain during the reaction excluded the possibility that Na_2_He is bound by weak interactions such as vdW. On the other hand, electronic structure analysis did not show any evidence that He formed chemical bonds with neighboring Na atoms, which immediately give rise to a question: how can He form a stable compound without forming chemical bonds (Miao, 2017)?

The answer to this paradox lies in the unusual behavior of electrons in Na under pressure. At pressures higher than 200 GPa, Na undergoes a structural transition and becomes transparent due to the presence of a large band-gap (Ma et al., 2009). In this double-hexagonal closed-packed structure, the valence electrons of Na detach from all the Na atoms and locate at the interstitial sites and play the role of anions. Electron analysis showed that Na_2_He is also an HPE (Dong et al., 2017), although Na atoms form a simple cubic lattice in Na_2_He. Therefore, the reaction can be viewed as the insertion of He into the Na_2_E ionic compound. Indeed, soon after the discovery of Na_2_He, the reaction of He with several other ionic compounds such as Na_2_O (Dong et al., 2017), Na_2_S (Gao et al., 2019), H_2_O (Liu et al., 2015), etc., have been predicted by DFT calculations. Similar to Na_2_He, He does not form chemical bonds with neighboring atoms in these compounds.

**Figure 2:**
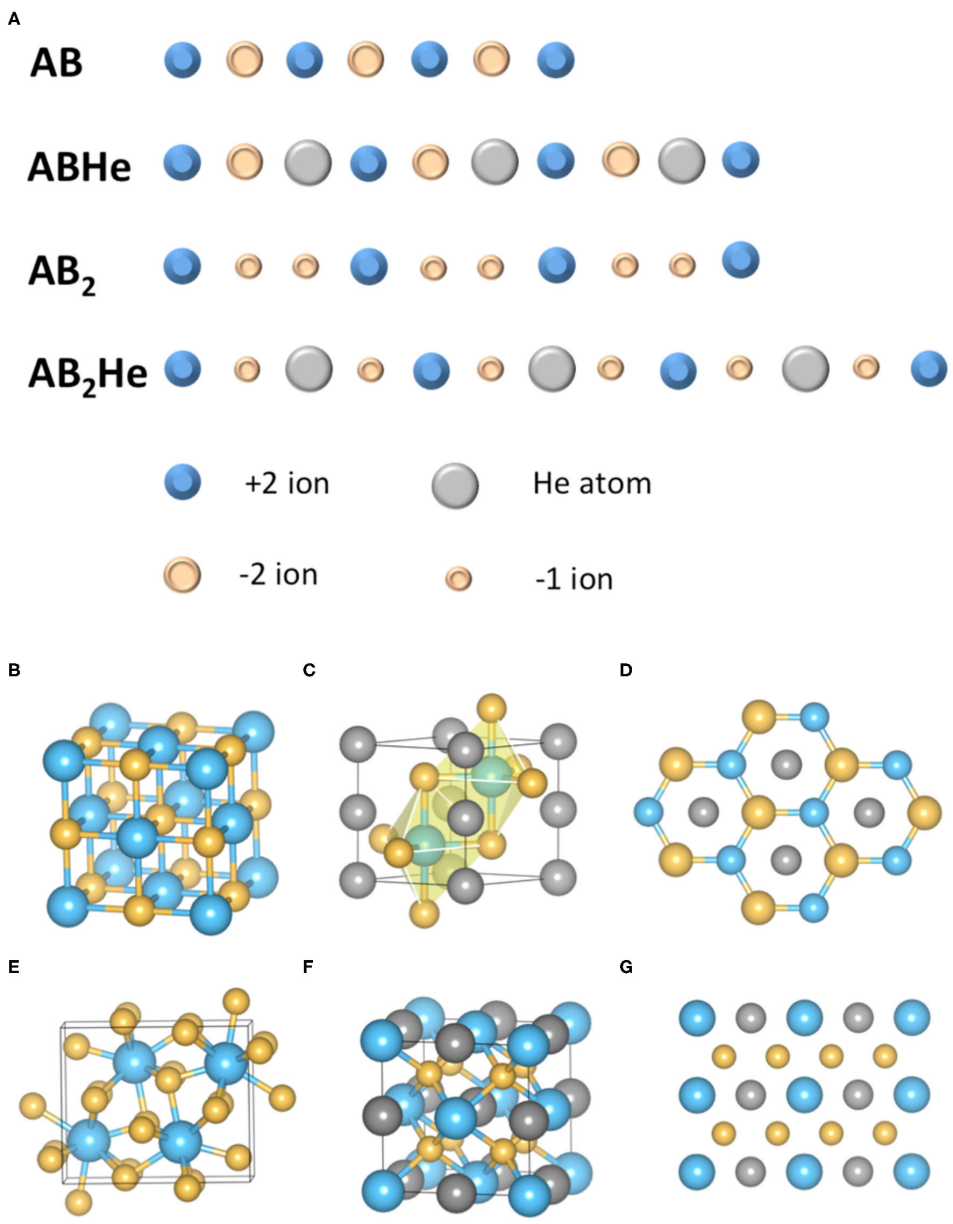
Mechanism of He insertion reaction with ionic compounds. **(A)** The schematics of He insertions into AB and AB_2_ types of ionic compounds. The Madelung energy increases in the former case whereas decreases in the latter case; **(B)** The rocksalt structure of MgO under high pressure; **(C)** The lowest energy structure of conceived compound MgOHe; **(D)** The view from (001) direction of MgOHe structure, showing that He chains are inserted between Mg and O atoms; **(E)** The PbCl_2_ structure of MgF_2_ under pressure; **(F)** The structure of stable MgF_2_He compound; **(G)** The (110) plane of MgF_2_He structure. The He atoms are inserted in between the neighboring F atoms, which further demonstrated the proposed mechanism. The blue, yellow and grey balls represent Mg, O (or F) and He atoms in **(B–G)**.

The driving force of He insertion reactions is electrostatic (Liu et al., 2018; Bai et al., 2019). The key point is that all the above ionic compounds involved in He insertion possess unequal numbers of cations and anions, although the overall charge is neutral. The mechanism can be explained more easily by a one-dimensional model (Figure 2A) (Liu et al., 2018). While reacting with the AB type of ionic compounds, He needs to be inserted in between two ions with opposite charges and therefore increase the electrostatic (Madelung) energy. In contrast, if the ionic compound is an A_2_B (or AB_2_) type, He atoms can choose to stay in between two ions (such as A^+^) with the same charge and therefore lower the Madelung energy. The two A^+^ ions repel each other, but are forced to stay close by external pressure. The insertion of the He in between two A+ ions alleviates this pressure effect, therefore the insertion of He in this type of ionic compounds becomes favored under increasing pressure. Thus, the reaction does not involve the formation of any local chemical bond, i.e., He can react with ionic compounds while keeping its chemical inertness (nobility). This mechanism has been demonstrated by rigorous energy analysis for He insertion into MgO (AB type) and MgF_2_ (AB_2_ type) compounds (Figures 2B–G) (Liu et al., 2018).

## Conclusions and Perspectives

Many recent simulations and experiments showed that noble gases could have very rich chemistry under high pressure. The types of chemical roles and interactions include electron donors (being oxidizing), electron acceptors (being reduced), NG–NG covalent bonds, noble gas bonds, and reliever of repulsive electrostatic interactions. A major effect of pressure is the change of the energies of the atomic orbitals. Although the energies of all local orbitals increase under higher pressure, the effect is more significant to those orbitals with lower principal quantum numbers and higher angular momenta, especially to those having no corresponding core orbitals such as 2p and 3d. As a result of this orbital energy reordering, the electrons are redistributed in different quantum orbitals under pressure. If the energy of the valence np orbital of a noble gas (5p for Xe, 4p for Kr) is close to or higher than the energies of the valence orbitals of an oxidant element such as F, O, or Fe, the noble gas will be oxidized. Conversely, if the unoccupied orbitals of the noble gases such as 5d for Xe become lower in energy than the orbitals of reductants such as Li or Mg, the noble gases will be reduced and become anionic.

The future of the exotic chemistry of noble gases as well as the high-pressure study rely on three signs of progress, the experimental methods that allow us to study the chemistry of materials under higher pressure (>200 GPa), the computer power and the simulation algorithms that can enable us to explore the structures and stability of more complicated materials such as ternary and quaternary compounds, the conceptual framework that can help us in understanding and predicting the change of chemistry under pressure without full-scale calculations. Although the recent high-pressure studies have greatly advanced noble gas chemistry, many important questions still remain unanswered. What are the oxidation and reduction limits of noble gases? Can He or Ne be oxidized or reduced under pressure? Can high-pressure noble gas compounds become superconducting or topological? Can high-pressure noble gas bonding be recovered after releasing the pressure? The developments of theoretical, simulation, and experimental methods might help to answer these questions and extend the noble gas chemistry to an unexpected territory.

## Author Contributions

The author confirms being the sole contributor of this work and has approved it for publication.

## Conflict of Interest

The author declares that the research was conducted in the absence of any commercial or financial relationships that could be construed as a potential conflict of interest.
